# A waveform library for the study of probing and ingestion behaviors of *Culicoides sonorensis* using AC–DC electropenetrography

**DOI:** 10.1186/s13071-025-06899-5

**Published:** 2025-07-07

**Authors:** Anastasia M. W. Cooper, Cameron J. Osborne, Victoria Pickens, Kaitlin Pfeiffer, Samuel B. Jameson, Anderson Rodrigo da Silva, Kathryn E. Reif, Dana N. Mitzel, Kristopher Silver

**Affiliations:** 1 Kansas State University, Manhattan, KS USA; 2 Auburn University, Auburn, AL USA; 3 Tulane University, New Orleans, LA USA; 4 Goiano Federal Institute, Urutai, GO Brazil; 5 National Bio and Agro-Defense Facility, Manhattan, KS USA

**Keywords:** Electropenetrography, EPG, Biting midges, *Culicoides sonorensis*, Behavior, Humans, Video recording, Ceratopogonidae, Blood feeding, Waveform

## Abstract

**Background:**

*Culicoides sonorensis* biting midges transmit arboviruses that negatively affect animal welfare and production in ruminant livestock operations. However, little is known about the probing and ingestion (i.e., biting) behaviors that occur inside host tissues, even though these behaviors may directly affect pathogen acquisition and transmission. Electropenetrography (EPG) allows for indirect visualization and quantification of these behaviors by measuring the changes in electrical signals that arise during probing.

**Methods:**

Using an alternating current–direct current (AC–DC) electropenetrograph, a waveform library for *C. sonorensis* biting behaviors was constructed from recordings of 70 adult females fed to repletion on human hands. The waveforms were characterized using each combination of four Ri levels (10^7^, 10^8^, 10^9^, and 10^10^ ohms) and two electrical current types (AC, DC). Five response variables related to the count and duration of the waveforms were analyzed for each waveform family, applying likelihood ratio tests and Tukey’s procedure to detect significant differences among the means of the eight treatment groups. The probability of transitioning between the waveform families was assessed on the basis of a frequency table of transition events, and multiple exact binomial tests were used to identify nonrandom transition events. Videography and interruption experiments were used to correlate behaviors with waveforms.

**Results:**

Waveforms generated by *C. sonorensis* included waveform families J/K (stylet penetration through the skin); L (types 1, 2, 3, 4, and 5; preparation of an ingestion site); M (types 1, 2, 3, 4, and 5; ingestion); N (types 1 and 2; an unknown behavior that may be a resting phase); and W (withdrawal). The waveforms generally occurred in that order, sometimes with multiple transitions between L, M, and N. Significant differences in the number of waveform events by insect for J, K, and L were observed between some DC treatment groups. The optimal setting for EPG recordings of *C. sonorensis* probing on human hands was an Ri level of 10^8^ ohms using an applied DC signal of 75 millivolts (mV).

**Conclusions:**

EPG uniquely enhances our understanding of *C. sonorensis* probing and ingestion behaviors, which will facilitate further exploration and guide the development of EPG procedures for other biting midges and telmophagous insects.

**Graphical Abstract:**

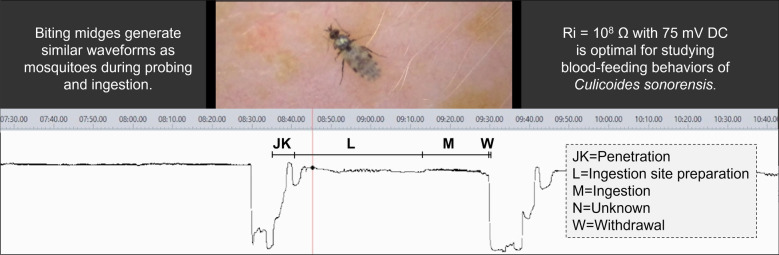

**Supplementary Information:**

The online version contains supplementary material available at 10.1186/s13071-025-06899-5.

## Background

*Culicoides* biting midges (Diptera: Ceratopogonidae) are minute hematophagous flies of worldwide medical, veterinary, and economic importance owing to their blood-feeding behaviors, which can stress hosts and facilitate pathogen transmission [1–3]. Adult female biting midges are pool feeders that sever dermal vessels with cutting mouthparts to obtain a blood meal from the hematoma that forms [4]. While there is a general understanding of how biting midge mouthparts function mechanistically, no studies have investigated plasticity in their probing and ingestion behaviors, even though variations in these behaviors could influence pathogen transmission and exacerbate host damage.

Recently, alternating current–direct current (AC–DC) electropenetrography (EPG) procedures were developed to characterize and quantify the unseen mouthpart movements and behaviors mosquitoes and ticks perform inside host tissues during blood meal acquisition. Investigations in *Aedes aegypti* [5] and *Culex tarsalis* [6] mosquitoes revealed differences in the duration, count, and probability of transitioning between waveforms corresponding to behaviors such as pre-probing, penetration of the skin, search for a blood vessel/ingestion site, ingestion, putative resting, and withdrawal of the mouthparts from the skin. In addition, infection of *Ae. aegypti* or a murine host with Dengue virus significantly altered the duration, count, and probability of transitioning between many of these behaviors, demonstrating that EPG can be used to quantify the effects of pathogens on cryptic blood-feeding behaviors [7]. Comparison of the early stages of slow-phase feeding in *Dermacentor variabilis* and *Amblyomma americanum* ticks additionally revealed variation in putative ingestion, salivation, and resting behaviors between the two species [8]. Together, these studies illustrate the value of EPG for investigating plasticity in probing and ingestion behaviors of blood-feeding arthropods. However, this technique has yet to be applied to biting midges, which are much smaller in size and have considerably different mouthparts, feeding strategies, and mechanisms of probing (i.e., biting) and ingestion than ticks and mosquitoes [9]. Owing to these differences, biting midges are likely to generate different waveforms corresponding to different probing and ingestion behaviors, as well as require different wiring procedures.

In North America, *Culicoides sonorensis* Wirth and Jones is the most abundant species of biting midge and a key vector for Bluetongue virus, epizootic hemorrhagic disease virus, and vesicular stomatitis virus [10]. Owing to its ability to transmit numerous pathogens and be laboratory-reared, *C. sonorensis* is a model species for *Culicoides* research [11]. The objectives of this study were to (1) identify waveform families and types generated by *C. sonorensis* probing on human hands; (2) determine the optimal settings for studying these waveforms; (3) describe the probability of transitioning between the waveforms; (4) infer the electrical origins of the waveforms and generate hypotheses about their biological meanings; and (5) link waveforms with observable behaviors. To accomplish these objectives, *C. sonorensis* blood-feeding on hands was recorded at four input resistance (Ri) levels and two current types using an AC–DC electropenetrograph. Five response variables related to the count and duration of the waveforms were analyzed, and the probability of transitioning between the waveform families was assessed. In addition, videography and interruption experiments were used to gain insight into the biological activities corresponding to each waveform.

## Methods

### Insects

**Fig. 1:**
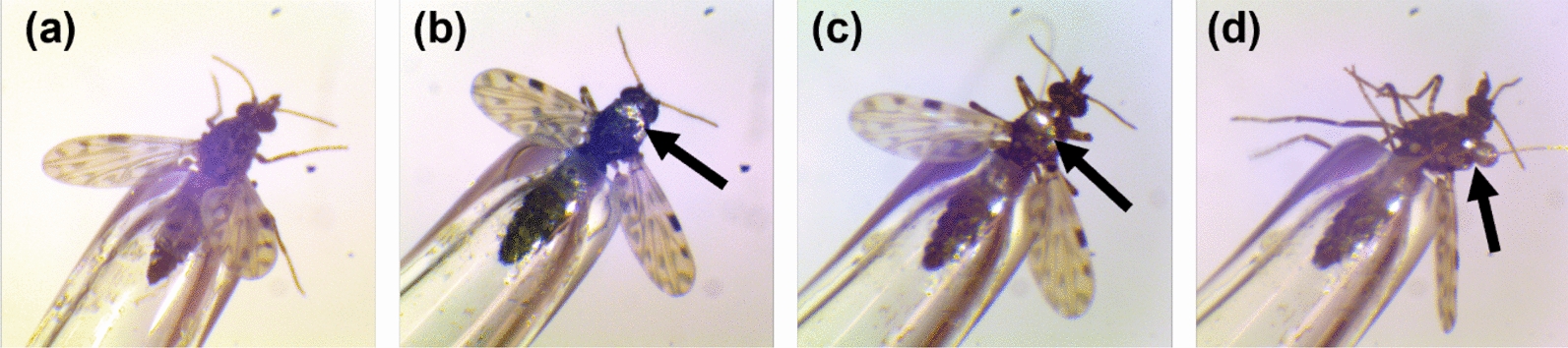
Photographs of electrode attachment to *Culicoides sonorensis*. The abdomen of an anesthetized biting midge was aspirated into a fired glass pipette (**a**). Silver glue was applied to the pronotum with a minutien pin (**b**). A gold wire with a ball of silver glue at the end was adhered to the silver spot of glue on the pronotum (**c**). The side view of the tethered midge held in the aspirator (**d**). Arrows indicate the location of the applied silver glue and glue ball

*Culicoides sonorensis* (AK strain) were obtained from the US Department of Agriculture-Agricultural Research Service (USDA-ARS) (Manhattan, KS) colony, which originated from a colony established in 1973 from material collected in Owyhee County, ID, USA [12]. Pathogen-free, adult female, blood-naïve *C. sonorensis* (1–16 days post-eclosion; Additional File 1: Supplementary Fig. S1) were prepared for EPG following Cooper et al. [6], with minor modifications to the silver glue formulation, gold wire diameter, and wire attachment procedure. A recording electrode (described in [5, 6, 13]) was made using a 3-cm long 0.0125-mm diameter 99.99% pure gold wire (Goodfelow, Pittsburgh, PA) and silver glue (recipe from [6]) with the addition of 1 µl of Triton X-100 to increase adhesion to the waxy cuticle. To form a glue ball for later attachment, the free end of the gold wire was dipped in silver glue (~15 times). For tethering, *C. sonorensis* that had been fasted for 8–12 h were anesthetized with CO_2_ gas until all movement ceased. Anesthetized midges were held by the abdomen using an insect aspirator fashioned from a modified Pasteur pipette under a dissection microscope for wiring (Fig. 1a). A thin layer of silver glue was applied to the pronotum using a minutien pin (Fig. 1b), followed by dipping the glue ball of the electrode into silver glue and immediately attaching the ball to the glue spot on the pronotum with a gentle rocking motion until the adhesion spot formed a bell shape (Fig. 1c, d). The wire was held in place until the glue dried, then tethered midges were placed in a plastic storage container and allowed to recover in an environmental chamber (2000 lux (lx); 26 °C, and > 60% RH) for 4–6 h before use.

### Human hosts

Humans were the hosts for all *C. sonorensis* experiments with approval from the Institutional Review Board for Human Subjects at Kansas State University (Manhattan, KS, USA; IRB no. 10729). During EPG sessions, the host would sit with their arm inside the Faraday cage and clasp a substrate voltage probe (i.e., reference electrode) in their hand, through which 50–150 mV alternating current (AC) or direct current (DC) substrate voltage was applied. While a tethered midge probed on their hand, the host observed it with a 5× hand lens and verbally reported their observations.

### EPG recordings

**Fig. 2:**
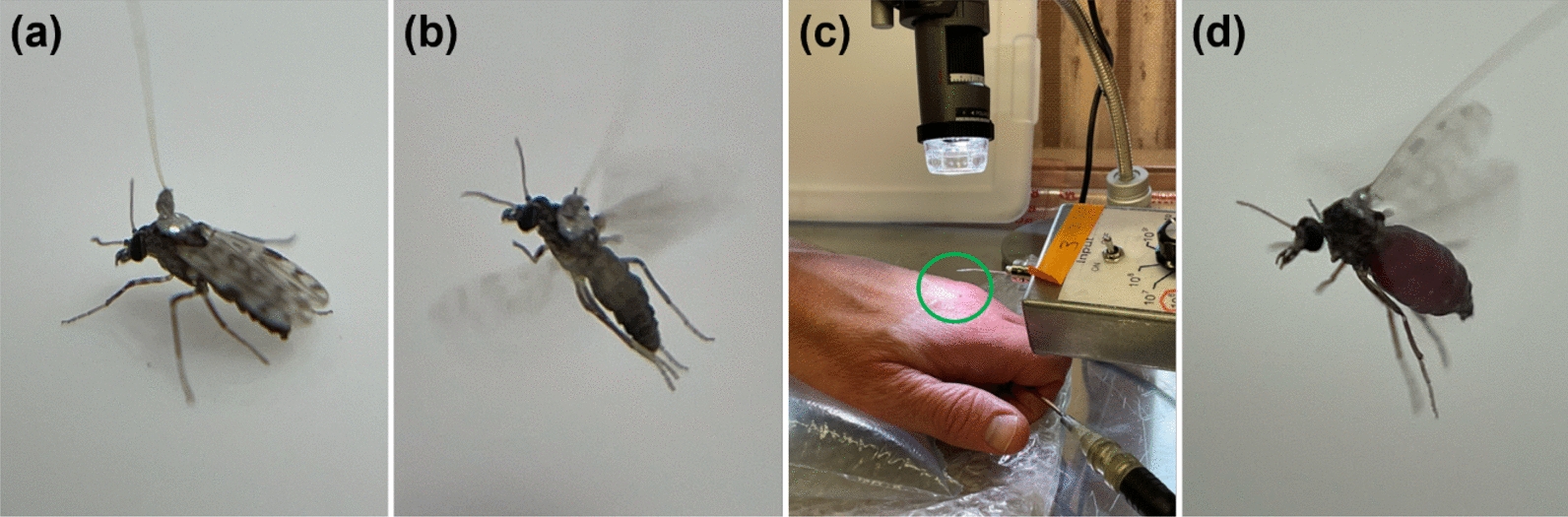
Photographs of *Culicoides sonorensis* prepared for electropenetrography (EPG). Close-ups of a tethered *C. sonorensis* standing (**a**) and flying (**b**). A tethered *C. sonorensis* (circled in green) attached to the head stage amplifier and standing under the digital microscope on a human hand while the human grasps the substrate voltage probe (**c**). An engorged *C. sonorensis* attempting to fly after EPG (**d**)

Individual midges were recorded probing on hands using a four-channel AC–DC electropenetrograph (EPG Technologies, Gainesville, FL, USA) and DATAQ Instruments Hardware Manager software (Dataq Instruments, Akron, OH, USA) as previously described [6, 13]. Eight to ten replicates were recorded for each combination of four Ri levels (10^7^, 10^8^, 10^9^, and 10^10^ ohms (Ω)) and two current types (AC, DC) using the substrate voltages and gain ranges specified (Additional File 1; Supplementary Table S1) so that the electrical origins of the waveforms and optimal EPG settings could be identified by comparing the appearance of the waveforms across the different settings [5, 6]. EPG sessions were conducted as detailed [6, 13], and the same criteria for the inclusion of data were used. In short, tethered *C. sonorensis* were individually connected to the head stage amplifier of the EPG and allowed access to the hand holding the substrate voltage probe, while exposed to fluorescent overhead lighting as well as indirect sunlight from an external window in the same location (i.e., laboratory benchtop) between 1 p.m. and 4 p.m. during each recording session. To be included in the dataset, the midges had to fly and walk normally, not detach from the wire, initiate probing within 10 min, feed to repletion, and produce interpretable waveforms (Fig. 2).

### Correlation of waveforms with behaviors

Observations reported by the hosts regarding midge movements, mouthpart locations, changes in appearance, and sensations felt were recorded as timestamped notes in the Hardware Manager software for correlation of the observations with waveforms. In addition, a subset of midges were video-recorded during EPG using a Dino-Lite Edge Digital Microscope (Dunwell Tech, Torrance, CA, USA) placed inside the Faraday cage for more detailed observation. Videography, synchronization with waveform data, scoring, analysis, and preparation for publication were performed as previously described [6].

To verify which waveforms were correlated with blood ingestion, interruption experiments were performed as previously published [6], where hosts withdrew their hands during the expression of different waveforms, and the midges were dissected to determine the presence of blood in the gut. All interruption experiments were performed at input resistance (Ri) = 10^9^ Ω using an applied AC signal of 50 mV.

### Measurement and analysis of waveforms

Waveform recordings (WDQ files) were reviewed using WinDaq Waveform Browser software (Dataq Instruments) as described [5, 6] for the identification and naming of the waveform families and types. Waveform families were scored, and the data were exported as TXT files following Cooper et al. [6]. The duration of each probe was defined at the start of waveform J to the time of withdrawal (W).

The average repetition rate (i.e., frequency) of the peaks in each waveform were estimated by averaging manual peak counts from at least one 1 s interval within each event of each waveform family or type in every probe. Longer waveform events were split into multiple events to exclude abnormal regions or regions of uncertain identity and ensure that a 1 s interval was counted at least once every 60 s. The 1 s interval(s) were randomly selected using a Random Integer Generator tool (Randomness and Integrity Services Ltd.; random.org). If more than one waveform type fell within the selected 1 s interval, the interval was shifted by 1–2 s to ensure the counted region fell within the event under consideration.

### Statistical analysis

To assess differences between the eight treatment groups (i.e., AC 10^7^, AC 10^8^, AC 10^9^, AC 10^10^, DC 10^7^, DC 10^8^, DC 10^9^, and DC 10^10^), TXT files corresponding to each WDQ file were uploaded directly into Insect Feeding Behavior Statistics (INFEST) 2.0 ([14]; https://arsilva.shinyapps.io/infest/) for calculation and statistical analysis of five response variables for each waveform family (J, K, L, M, and N): the waveform duration in seconds by insect (WDi), the number of waveform events by insect (NWEi), the waveform duration in seconds per event by insect (WDEi), the duration in seconds of the first event of the specified waveform (DurFrs), and the mean number of events to the first event of the specified waveform (NumPrFrs). Extreme values in the dataset were identified on the basis of the Weibull distribution fitted to each variable considering the quantile limits 0.01 and 0.99 [15]. Outlier candidates were removed after considering the impact on the sample size of the group, and the number of outlier candidates in the group. Next, General Additive Models for Location, Scale, and Shape (GAMLSS) [16] were fitted to the count and duration variables, using treatment as covariates to model both the mean and dispersion of the treatment levels. Akaike’s information criterion (AIC) was used to select the distribution model for each response variable. The goodness of fit of models was evaluated on the basis of root mean squared error (RMSE) and graphical diagnostics on residuals using worm plots. The likelihood ratio test was applied to detect significant effects of treatments. Inferences with the marginal means of groups were performed considering asymptotic normality through pairwise comparisons using *z*-tests with a 5% nominal significance level (*α* = 0.05) on the basis of Tukey’s method for *p*-value adjustment. “Per insect” estimates (i.e., the mean and standard error for all midges in each treatment group, denoted by an uppercase “I”) were generated during statistical analysis for the five “by insect” response variables listed above (i.e., calculated for each midge individually, denoted by a lowercase “i”) and are reported in the results.

A kinetogram showing the probability of transitioning between the waveform families was also generated by INFEST 2.0 on the basis of frequency tables of transition events. Multiple exact binomial tests with a 5% nominal significance level (*α* = 0.05) were used to identify nonrandom transition events. To show the probability of each insect feeding to repletion or probing again, the initial nonprobing (NP) event at the beginning of each recording (which preceded the first probe) was relabeled in each TXT file as “NPi,” the last occurrence of NP after the final or singular probe as “NPf,” and all occurrences of NP between probes as “NPm” (i.e., NP initial, middle, and final).

## Results

### Overview of dataset

A total of 245 EPG recordings were made using 234 biting midges during 27 EPG sessions conducted between May and July 2022. In total, 77 midges (32.9%) probed, and 76 (32.5%) fed to repletion on the offered hand. Of these 76 EPG recordings, 70 had interpretable waveforms suitable for waveform library construction. Of the 70 midges that fed to repletion and had usable waveforms, 19 individuals (27.9%) required multiple (up to five) probes before feeding to repletion. Thus, 106 probes from 70 biting midges were used to construct the waveform library (Additional File 1: Supplementary Table S2). Owing to peaking out (defined in [13]) during K and low resolution from suboptimal gain settings, 59 (containing 87 probes) were suitable for estimation of peak frequencies (Table 1), and 68 of the 70 EPG recordings (containing a total of 104 midge probes) were suitable for statistical analysis (Table 2), respectively. These recordings were made on seven hosts (four females and three males between the ages of 23 and 44 years old; Supplementary Fig. S1a). Owing to host availability, 47.1% of the recordings were made on one volunteer’s hand (Additional File 1: Supplementary Fig. S2a). All volunteers reported standard reactions to the bites and could not feel the applied voltage. More midges were required to record 8–10 probes to repletion at AC 10^9^ than the other settings (Additional File: Supplementary Fig. S2b). Of the 35 videos recorded during EPG, 8 were suitable for synchronization with waveform data, scoring, and behavioral analysis. An additional 33 interrupted midge probes were used to investigate when blood ingestion occurred (Table 3).

**Table 1:** Characteristics of waveforms generated by *Culicoides sonorensis* probing on human hands

Phase	Family	Type	Avg. peak frequency ± SE (Hz)	Optimal Ri levels (Ω)	% of probes with waveform	Electrical origin	Additional description
Penetration	J	na	5.81 ± 0.35	10^9^, 10^10^	100%	Emf dominated, some R	Spike at the onset is an emf component
	K	na	5.55 ± 0.31	10^8^, 10^9^	100%	Mixed emf + R	Sharp peak(s) at beginning of plateau is an R component
Ingestion site preparation	L	L1	6.07 ± 0.19	10^8^, 10^9^	86.2%	Mixed emf + R	Low-amplitude rhythmic peaks or humps
		L2	5.64 ± 0.15	10^8^, 10^9^	89.7%	Mixed emf + R	Larger amplitude, erratic, jagged peaks with an irregular peak frequency
		L3	6.73 ± 0.21	10^8^, 10^9^	40.2%	Mixed emf + R	Mix of plateaus and downward-going peaks
		L4	1.07 ± 0.14	10^8^, 10^9^	79.3%	Mixed emf + R	Mostly flat, with only a few small peaks or bumps
		L5	12.67 ± 0.32	10^7^, 10^8^	52.9%	R dominated, some emf	Very low amplitude irregular peaks; best visualized with high WinDaq gains (64–128×)
Ingestion	M	M1	8.08 ± 0.10	10^8^, 10^9^	82.8%*	Mixed emf + R	Rhythmic sine waves
		M2	5.96 ± 0.21	10^8^, 10^9^	49.4%*	Mixed emf + R	Jagged irregular sine wave-like peaks
		M3	8.00 ± 0.55	na**	3.4%*	na**	Mix of peaks and plateaus with small peaks at the top of the plateaus
		M4	1.82 ± 0.24	10^8^, 10^9^	39.1%*	Mixed emf + R	Mostly flat with occasional peaks
		M5	5.66 ± 0.26	10^7^, 10^8^	37.9%	R dominated, some emf	Short repeats of M1, M2, M3, and M4 with a characteristic oscillating appearance from low- and high-frequency regions of different amplitudes***
Unknown	N	N1	2.78 ± 0.37	na**	13.8%	na**	Irregularly shaped, skewed peaks
		N2	0.71 ± 0.10	10^8^, 10^9^	42.5%	Mixed emf + R	Relatively flat with very few peaks
Withdraw	W	na	na	10^9^, 10^10^	100%	Emf dominated, some R	

^*^Does not include occurrences observed during M5

^**^Insufficient observation to determine

^***^The peaks in M5 were more prominent at lower Ri levels and usually comprised short repeats of high-frequency types (M1, M2, and M3), whereas, at higher Ri levels, M4 was present between the high-frequency types

*AC* alternating current, *Avg.* average, *DC* direct current, *emf* electromotive forces, *Hz* hertz, *na* not applicable, *R* biological resistance, *Ri* input resistance, *SE* standard error, *Ω* ohms

**Table 2:** Descriptive statistics and summary of multiple comparisons of means of five common EPG variables

WF	Var	AC 10^7^	AC 10^8^	AC 10^9^	AC 10^10^	DC 10^7^
J	WDi	10.37 ± 2.54 [8]	5.07 ± 1.24 [8]	9.46 ± 2.07 [10]	8.64 ± 2.12 [8]	11.55 ± 2.83 [8]
	NWEi	1.50 ± 0.25 ab [8]	1.25 ± 0.19 ab [8]	1.70 ± 0.27 ab [10]	1.62 ± 0.28 ab [8]	1.75 ± 0.31 ab [8]
	WDEi	7.30 ± 2.51 [8]	4.24 ± 1.03 [8]	6.37 ± 1.38 [10]	6.95 ± 2.48 [8]	6.01 ± 0.84 [8]
	DurFrs	7.36 ± 2.12 [8]	4.23 ± 0.92 [8]	6.41 ± 1.54 [10]	6.87 ± 1.91 [8]	5.29 ± 1.29 [8]
	NumPrFrs	1.00 ± 0.00 [8]	1.00 ± 0.00 [8]	1.00 ± 0.00 [10]	1.00 ± 0.00 [8]	1.00 ± 0.00 [8]
K	WDi	3.99 ± 0.95 [8]	2.42 ± 0.44 [8]	2.88 ± 0.55 [9]	3.51 ± 0.78 [8]	4.68 ± 1.20 [8]
	NWEi	1.50 ± 0.25 ab [8]	1.25 ± 0.19 ab [8]	1.70 ± 0.26 ab [10]	1.62 ± 0.28 ab [8]	1.75 ± 0.31 ab [8]
	WDEi	2.74 ± 0.43 [8]	2.06 ± 0.28 [8]	2.37 ± 0.31 [10]	2.20 ± 0.31 [8]	2.62 ± 0.40 [8]
	DurFrs	2.90 ± 0.48 [8]	2.08 ± 0.29 [8]	2.12 ± 0.27 [10]	2.34 ± 0.35 [8]	3.22 ± 0.56 [8]
	NumPrFrs	2.00 ± 0.00 [8]	2.00 ± 0.00 [8]	2.00 ± 0.00 [10]	2.00 ± 0.00 [8]	2.00 ± 0.00 [8]
L	WDi	133.9 ± 41.44 [8]	83.0 ± 20.25 [8]	108.8 ± 27.17 [10]	84.2 ± 20.68 [8]	99.7 ± 28.50 [7]
	NWEi	1.62 ± 0.27 ab [8]	1.25 ± 0.19 ab [8]	1.70 ± 0.26 ab [10]	1.88 ± 0.34 ab [8]	1.88 ± 0.34 ab [8]
	WDEi	81.3 ± 17.76 [8]	66.7 ± 13.19 [8]	64.7 ± 11.29 [10]	42.7 ± 6.75 [8]	74.1 ± 15.46 [8]
	DurFrs	93.7 ± 17.29 [8]	73.9 ± 13.64 [8]	70.6 ± 11.64 [10]	47.5 ± 8.77 [8]	65.3 ± 12.04 [8]
	NumPrFrs	3.00 ± 0.00 [8]	3.00 ± 0.00 [8]	3.00 ± 0.00 [10]	3.00 ± 0.00 [8]	3.00 ± 0.00 [8]
M	WDi	369 ± 47.6 [8]	318 ± 38.1 [8]	333 ± 36.5 [10]	287 ± 32.6 [8]	371 ± 48.0 [8]
	NWEi	1.25 ± 0.16 [8]	1.38 ± 0.18 [8]	1.40 ± 0.17 [10]	1.62 ± 0.23 [8]	1.50 ± 0.21 [8]
	WDEi	328 ± 56.8 [8]	264 ± 45.9 [8]	294 ± 45.5 [10]	227 ± 39.3 [8]	287 ± 49.9 [8]
	DurFrs	316 ± 86.4 [8]	329 ± 39.3 [7]	324 ± 51.7 [10]	318 ± 21.7 [7]	283 ± 74.2 [8]
	NumPrFrs	5.25 ± 0.57 [8]	4.00 ± 0.38 [8]	4.00 ± 0.35 [9]	4.62 ± 0.47 [8]	5.25 ± 0.57 [8]
*N*	WDi	193.0 ± 67.8 [6]	189.5 ± 66.6 [6]	96.2 ± 33.8 [6]	67.1 ± 23.6 [6]	79.0 ± 34.0 [4]
	NWEi	1.00 ± 0.05 [7]	1.17 ± 0.07 [6]	1.00 ± 0.05 [6]	1.14 ± 0.06 [7]	1.00 ± 0.06 [4]
	WDEi	193.0 ± 66.9 [6]	168.4 ± 58.4 [6]	96.2 ± 33.3 [6]	55.0 ± 19.1 [6]	79.0 ± 33.6 [4]
	DurFrs	193.0 ± 123.5 [6]	166.0 ± 98.5 [6]	96.2 ± 43.5 [6]	48.5 ± 15.5 [6]	79.0 ± 39.6 [4]
	NumPrFrs	7.57 ± 1.17 [7]	6.00 ± 0.89 [6]	9.00 ± 1.64 [6]	6.29 ± 0.89 [7]	9.75 ± 2.27 [4]

DC 10^8^	DC 10^9^	DC 10^10^	Distribution	Stats
9.63 ± 2.22 [9]	8.51 ± 1.97 [9]	6.25 ± 1.53 [8]	Gamma	LTR_(7)_ = 7.71, *P* = 0.359
2.11 ± 0.39 b [9]	1.22 ± 0.17 ab [9]	1.00 ± 0.13 a [8]	Inverse Gaussian	LTR_(7)_ = 14.38, *P* = 0.045*
4.72 ± 0.45 [9]	6.77 ± 1.78 [9]	6.25 ± 1.00 [8]	Inverse Gaussian†	LTR_(7)_ = 6.08, *P* = 0.531
4.08 ± 0.83 [9]	6.86 ± 1.80 [9]	6.25 ± 1.66 [8]	Inverse Gaussian	LTR_(7)_ = 5.61, *P* = 0.586
1.00 ± 0.00 [9]	1.00 ± 0.00 [9]	1.00 ± 0.00 [8]	na	na
4.92 ± 1.22 [9]	2.61 ± 0.47 [9]	2.21 ± 0.39 [8]	Inverse Gaussian	LTR^(7)^ = 13.30, *P* = 0.065
2.11 ± 0.39 b [9]	1.22 ± 0.17 a [9]	1.00 ± 0.13 a [8]	Inverse Gaussian	LTR_(7)_ = 14.38, *P* = 0.045*
2.39 ± 0.33 [9]	2.05 ± 0.26 [9]	2.21 ± 0.31 [8]	Zero adjusted IG	LTR_(7)_ = 3.80, *P* = 0.803
2.38 ± 0.34 [9]	2.08 ± 0.27 [9]	2.21 ± 0.32 [8]	Inverse Gaussian	LTR_(7)_ = 8.19, *P* = 0.317
2.00 ± 0.00 [9]	2.00 ± 0.00 [9]	2.00 ± 0.00 [8]	na	na
75.0 ± 17.38 [8]	49.0 ± 9.19 [8]	83.7 ± 20.48 [8]	Zero adjusted IG	LTR_(7)_ = 9.29, *P* = 0.232
2.22 ± 0.41 b [9]	1.22 ± 0.17 ab [9]	1.00 ± 0.13 a [8]	Inverse Gaussian	LTR_(7)_ = 17.13, *P* = 0.017*
48.3 ± 7.68 [9]	63.0 ± 11.43 [9]	83.7 ± 18.55 [8]	Inverse Gaussian	LTR_(7)_ = 10.03, *P* = 0.187
75.9 ± 14.00 [8]	43.0 ± 7.93 [8]	83.7 ± 15.43 [8]	Zero adj. Gamma	LTR_(7)_ = 112.81, *P* = 0.077
3.00 ± 0.00 [9]	3.00 ± 0.00 [9]	3.00 ± 0.00 [8]	na	na
363 ± 43.7 [9]	303 ± 33.4 [9]	343 ± 42.7 [8]	Inverse Gaussian	LTR_(7)_ = 4.23, *P* = 0.753
1.67 ± 0.23 [9]	1.11 ± 0.12 [9]	1.00 ± 0.11 [8]	Inverse Gaussian	LTR_(7)_ = 12.71, *P* = 0.080
242 ± 41.9 [8]	287 ± 47.0 [9]	343 ± 59.6 [8]	Zero adj. Gamma	LTR_(7)_ = 4.47, *P* = 0.724
235 ± 104.3 [9]	342 ± 46.7 [9]	382 ± 41.9 [8]	Weibull^†^	LTR_(7)_ = 2.89, *P* = 0.895
5.67 ± 0.60 [9]	5.11 ± 0.51 [9]	4.00 ± 0.38 [8]	Inverse Gaussian	LTR_(7)_ = 13.31, *P* = 0.065
125.0 ± 48.1 [5]	104.5 ± 40.2 [5]	170.5 ± 65.6 [5]	Zero adj. Gamma	LTR_(7)_ = 7.70, *P* = 0.360
1.00 ± 0.06 [5]	1.00 ± 0.6 [5]	1.00 ± 0.06 [5]	Zero adjusted IG	LTR_(7)_ = 10.03, *P* = 0.190
125.0 ± 47.5 [5]	104.5 ± 39.7 [5]	170.5 ± 64.8 [5]	Zero adj. Gamma	LTR_(7)_ = 8.71, *P* = 0.274
125.0 ± 70.6 [5]	104.5 ± 54.0 [5]	170.5 ± 112.4 [5]	Zero adjusted IG	LTR_(7)_ = 5.50, *P* = 0.599
7.40 ± 1.34 [6]	5.00 ± 0.75 [4]	5.00 ± 0.075 [5]	Inverse Gaussian	LTR_(7)_ = 12.55, *P* = 0.084

^*^Statistically significant *(P* < *0.05)*

^†^The treatment group factor was used as a covariate to model variances of groups

Descriptive statistics (mean ± standard error) and summary of multiple comparisons of means of five common EPG variables for each *Culicoides sonorensis* waveform family when recorded with different input resistor (Ri) levels and current type (I) combinations (i.e., groups). Results of likelihood ratio (LTR) tests for treatment groups are presented, including LTR test statistics, degrees of freedom in parentheses, and *P*-values. The probability distribution models selected (lowest AIC) for each response variable using GAMLSS are reported. Significant (*P* ≤ 0.05) differences between means were assessed using *z*-tests with Tukey’s procedure for pairwise comparisons, and means sharing the same letter across rows are not significantly different. The number in brackets indicates the number of insects performing each waveform in a comparison of 68 insects total, minus extreme outliers. *AC* alternating current, *DC* direct current, *DurFrs* duration of first event per insect, *EPG* electropenetrography, *GAMLSS* General Additive Models for Location, Scale, and Shape, *IG* Inverse Gaussian, *N* number of insects performing each waveform, *na* not applicable, *NumPrFrs* number of events to first event per insect, *NWEI* number of waveform events per insect, *SE* standard error, *Var.* variable, *WDEI* waveform duration in seconds per event per insect, *WDI* waveform duration in seconds per insect, *WF* waveform family

**Table 3:** Results from interruption experiments with *Culicoides sonorensis*

Interrupted waveform	Duration before waveform interrupted (s)	Blood: no blood
K	2.5–3.5	0:4
L	11.5–24	0:6
	32–86	0:7
M	6.5–11	5:0
	20–26.5	5:0
	28.5–50	6:0

The ratio of *Culicoides sonorensis* individuals with and without blood observed in the alimentary canal after interrupting probes at various time points during specific waveforms

### Overview of waveforms and transition probabilities

**Fig. 3:**
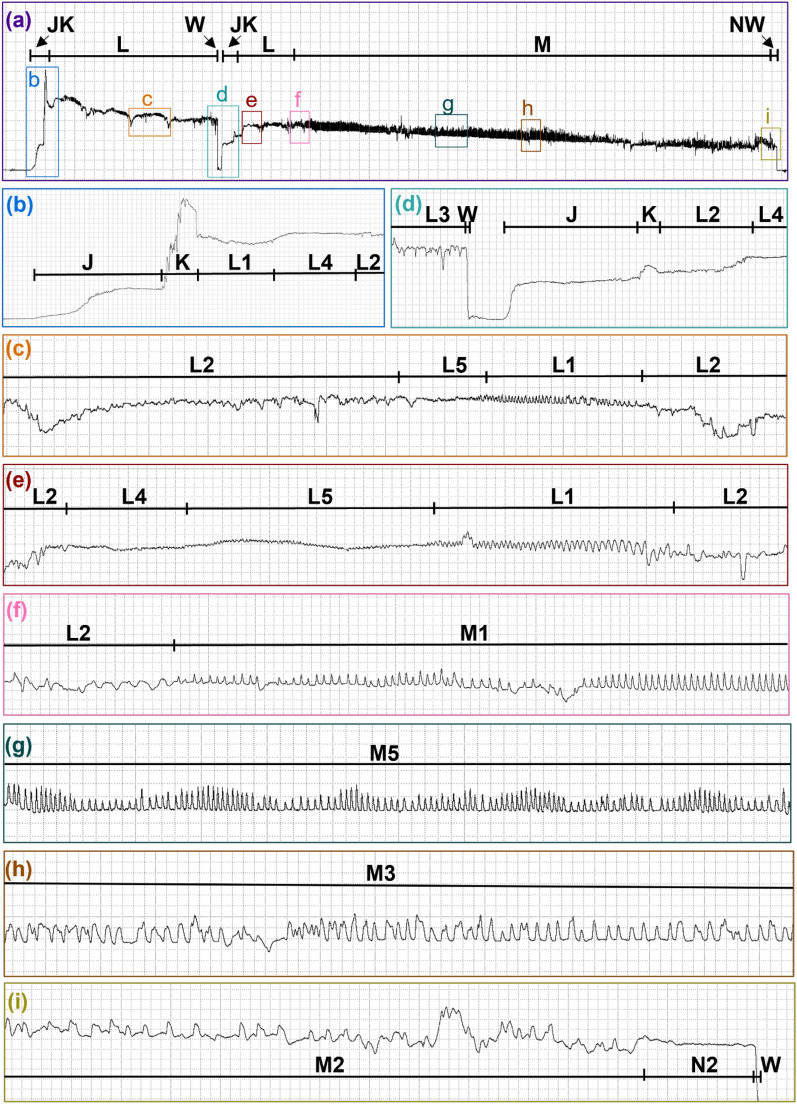
Representative waveforms recorded with AC and Ri = 10^7^. Representative waveforms from two *Culicoides sonorensis* probes on a human hand recorded with an input resistance of 10^7^ ohms using an applied AC signal of 150 mV. Family-level (J, K, L, M, N, and W) and type-level (L1, L2, L4, L5, M1, M2 M3, M5, and N2) waveform names are shown along the top of each panel. Lower panels show enlargements of select regions in panel **a**. Time scales and WinDaq gains are: 9.0 s per division (s/div), 32× (**a**); 0.4 s/div, 32× (**b**); 0.6 s/div, 64× (**c**); 0.6 s/div, 64× (**d**); 0.2 s/div, 64× (**e–f**); 0.4 s/div, 64× (**g**); and 0.2 s/div, 64× (**h–i**). Note that the position of J, K, and N is an approximation in panel **a**; see panels **b**,** d**, and **i** for exact positions

**Fig. 4:**
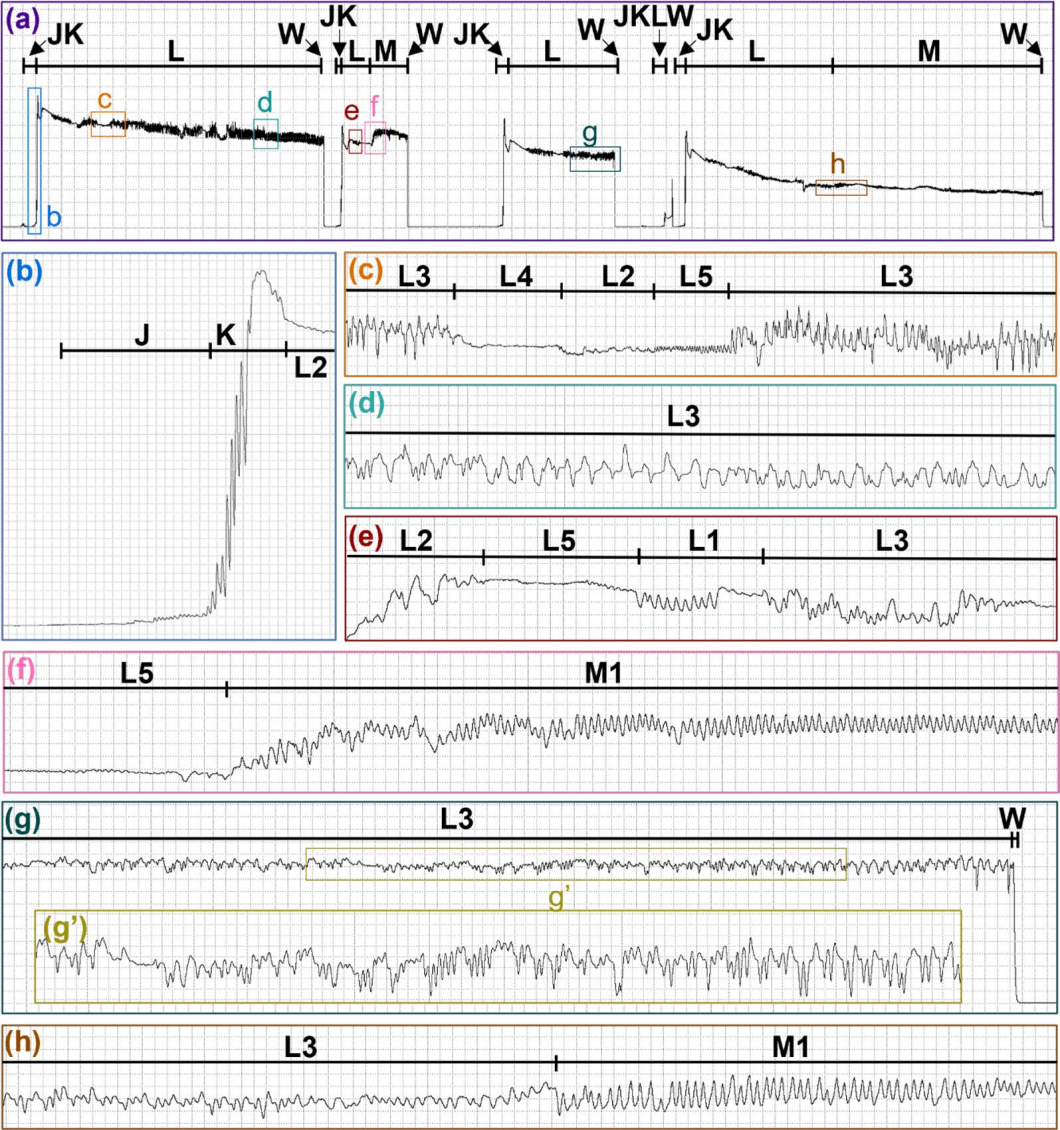
Representative waveforms recorded with DC and Ri=10^8^. Representative waveforms from five *Culicoides sonorensis* probes on a human hand recorded with an input resistance of 10^8^ ohms using an applied DC signal of 75 mV. Family-level (J, K, L, M, W) and type-level (L1, L2, L3, L4, L5, M1) waveform names are shown along the top of each panel. Lower panels show enlargements of select regions in panel **a**. Panel **g’** insert shows an enlargement of the specified region in panel **g**. Time scales and WinDaq gains are: 11 seconds per division (s/div), 4x (**a**); 0.2 s/div, 32x (**b**); 0.4 s/div, 64 x(**c**); 0.2 s/div, 32x (**d**-**e**); 0.4 s/div, 16x (**g**); 0.2 s/div, 64x (**g’**-**h**). Note that the position of J and K is an approximation in panel **a**. For the exact position, please see panel **b**

At the start of a probe, the measured current would rise slightly above the NP baseline before shooting up into a very brief plateau, and then decrease slightly as repetitive low amplitude peaks, plateaus, expanses, and sine waves occurred throughout the probe until the mouthparts were withdrawn and the voltage level immediately returned to NP (Additional File 2: Supplementary Video S1). These voltage changes during probing were divided into six waveform families (J, K, L, M, N, and W) and 12 types (L1, L2, L3, L4, L5, M1, M2, M3, M4, M5, N1, and N2), summarized in Table 1. Probe waveforms predominantly varied in appearance across Ri levels (Figs. 3, 4).

**Fig. 5:**
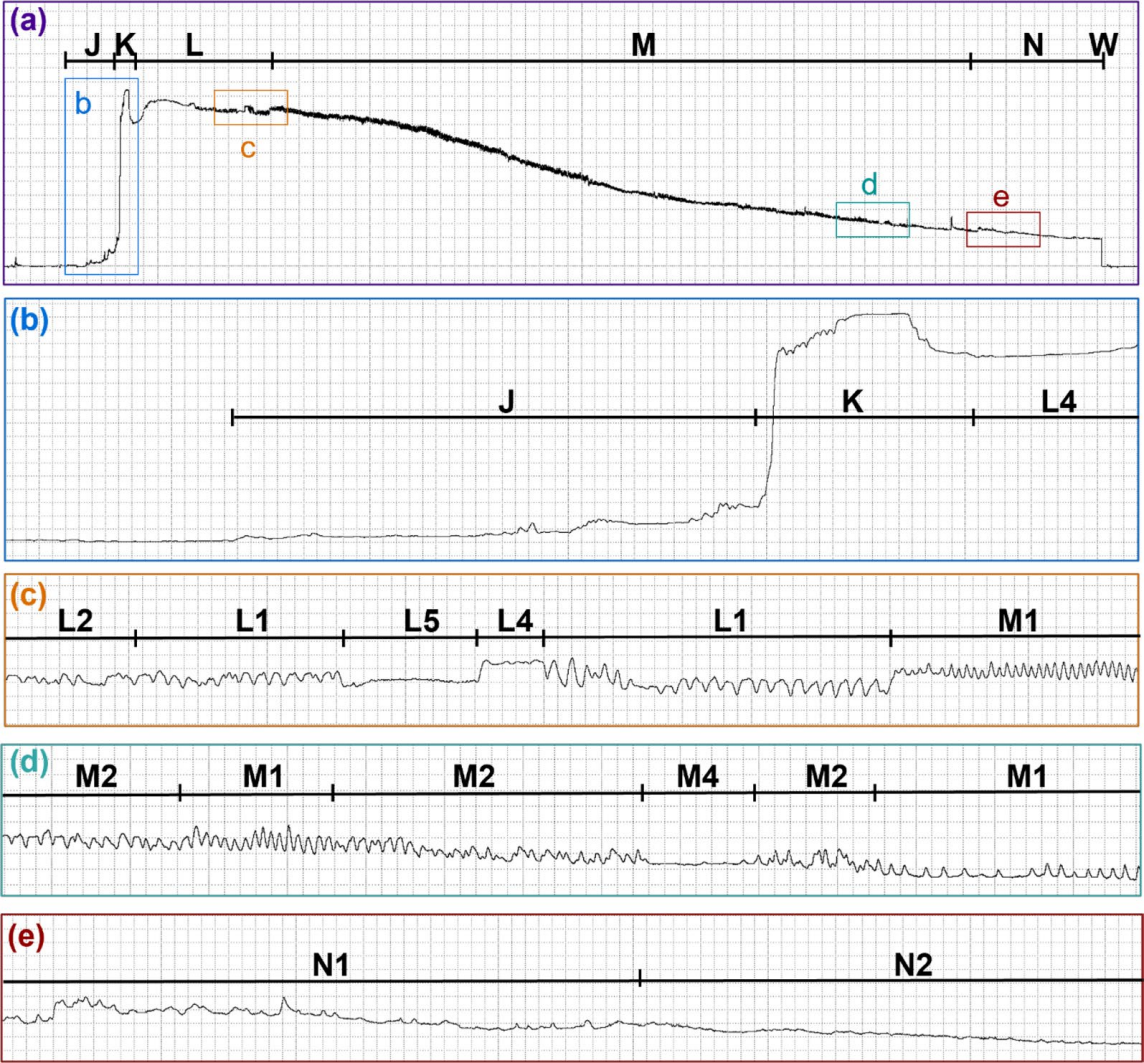
Representative waveforms recorded with DC and Ri=10^9^. Representative waveforms from a *Culicoides sonorensis* probe on a human hand recorded with an input resistance of 10^9^ ohms using an applied DC signal of 50 mV. Family-level (J, K, L, M, N, W) and type-level (L1, L2, L4, L5 M1, M2, M4, N1, N2) waveform names are shown along the top of each panel. Lower panels show enlargements of select regions in panel **a**. Time scales and WinDaq gains are: 3.4 seconds per division (s/div), 16x (**a**); 0.2 s/div, 16x (**b**); 0.2 s/div, 32 x (**c**); 0.2 s/div, 64x (**d**-**e**). Note that the position of J and K is an approximation in panel **a**; see panel **b** for exact positions

On average, *C. sonorensis* probed 1.46 times before feeding to repletion. Probes preceding the final probe (i.e., the probes before the last probe in the series) were shorter in duration (1.93 ± 0.25 min) than final/singular probes (8.14 ± 0.41 min). Probes preceding the final probe contained J, K, L, and W, with half containing M. Singular and final probes usually contained J, K, L, M, and W, but 70.6% of final/singular probes also included N, and one final probe (0.96%) lacked both M and N. For all scored probes (preceding, final, and singular), the probability of initially transitioning from NP to J was 1 (Fig. 5). After J, 100% of probes transitioned to K and then to L. From L, 86.5% of probes transitioned to M, with only 18.2% transitioning to W instead; however, this could have happened randomly, according to an exact binomial test (*P* = 0.083; Supplementary Table S3). After M, 39.4% of the probes transitioned to W, whereas 42.3% transitioned to N before W. However, 2.9% transitioned from N back to M before W, but this may have occurred by chance (*P* = 0.944), such as the 4.8% that transitioned from M to L (*P* = 0.993). After W, all the midges transitioned back to NP. The probability that the midge had fed to repletion after W and stopped probing was 0.654; however, there was a 3.5% chance that the midge would probe again before feeding to repletion.

### Waveform characterization and behavioral correlation

#### Family J

**Fig. 6:**
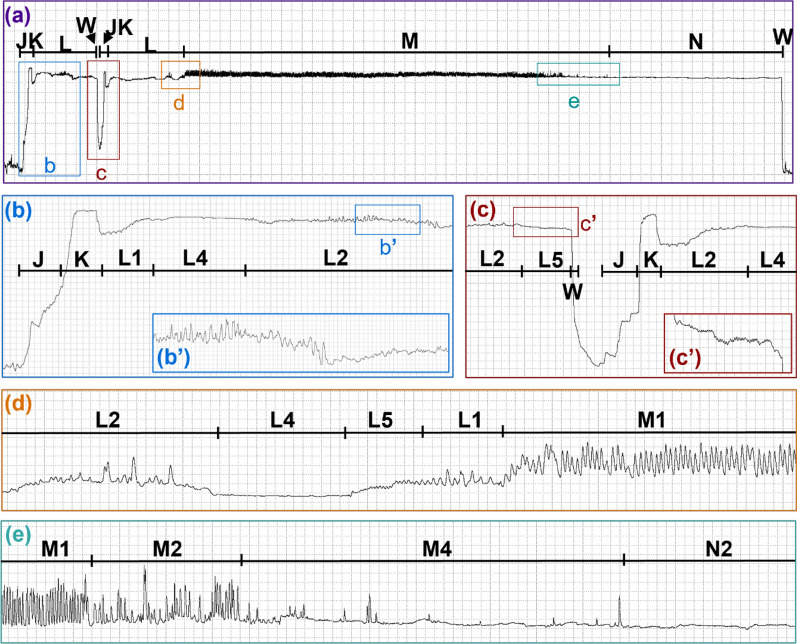
Representative waveforms recorded with AC and Ri=10^10^. Representative waveforms from two *Culicoides sonorensis* probes on a human hand recorded with an input resistance of 10^10^ Ohms using an applied AC signal of 50 mV. Family-level (J, K, L, M, N, W) and type-level (L1, L2, L4, L5, M1, M2, M4, N2) waveform names are shown along the top of each panel. Lower panels show enlargements of select regions in panel **a**. Panels **b’** and **c’** inserts show enlargements of the specified regions in panels **b** and **c**, respectively. Time scales and WinDaq gains are: 5.6 seconds per division (s/div), 4x (**a**); 0.4 s/div, 8x (**b**); 0.2 s/div, 64x (**b’**); 0.4 s/div, 8x (**c**); 0.2 s/div, 128x (**c’**); 0.2 s/div, 32x (**d**); 0.6 s/div, 64x (**e**). Note that the position of J and K is an approximation in panel **a**; see panels **b** and **c** for exact positions

J occurred immediately before the most prominent voltage spike at the beginning of the *C. sonorensis* probe and appeared as a slight to moderate voltage rise above the NP baseline, usually with a small spike at the onset (Figs. 3, 4, 6; Table 1). In the videos, J was correlated with the initial penetration of the host’s skin (Additional Files 2–6: Supplementary Videos S1–S5), at the onset of which spreading of the maxillary palps usually occurred, occasionally accompanied by subsequent head bobbing. Hosts were typically unaware of probe initiation but reported that locomotion had stopped.

No significant differences between treatment groups were detected for J, except in NWEI (Table 2). NWEI of J was estimated to be between 1.00 and 2.11, reflecting that 19 of the 64 midges probed multiple times before feeding to repletion. The likelihood ratio test indicated that the current type and Ri level combinations used to record the waveforms had a significant effect on the NWEI for J (LTR_(7)_ = 14.38, *P* = 0.045), and pairwise comparisons with *z*-tests showed that NWEI of J was 2.11 ± 0.477-fold higher when using DC 10^8^ Ω compared with DC 10^10^ Ω (*Z* = 3.31, *P* = 0.021). The WDEI and WDI indicated that, on average, each event (i.e., individual occurrence) of J was between 4.24 s and 7.30 s long, and the total amount of time each midge spent performing waveform J was between 5.07 and 10.37 s, respectively. DurFrs for J was very similar to the estimates for WDEI, indicating that the first occurrence of J was similar to the duration of subsequent occurrences in successive probes. NumPrFrs for J was exactly 1.00 because J always occurred at the beginning of every probe, which was always preceded by NP.

#### Family K

K appeared as a prominent voltage spike that plateaued before the signal dipped slightly (Figs. 3, 4, 6; Table 1). Most video observations indicated that the mouthparts were still penetrating the skin during K, but at least in one instance, the mouthparts appeared already fully embedded (Additional Files 2–6: Supplementary Video S1–S5). No blood was observed in the alimentary canal of midges interrupted and dissected during K (Table 3).

NEWI for K was the same as for J because K occurred once at the beginning of every probe (Table 2; same statistical output and interpretation as above) and always occurred immediately after J (NumPrFrs = 2.00). Estimates for WDEI and DurFrs were similar to each other, with each event of K on average 2.06–3.22 s in duration. WDI estimates indicated that each midge spent an average of 2.21–4.92 s performing K.

#### Family L

L usually occurred following K, with a pronounced dip in voltage that quickly leveled out to a slightly lower voltage level than K (Figs. 3, 4, 6), occasionally with additional voltage dips (Fig. 3a). A series of peaks, plateaus, and expanses were observed and divided into five waveform types: L1, L2, L3, L4, and L5 (Table 1).

NumPrFrs for waveform family L was 3.00, reflecting that all *C. sonorensis* probes contained L following K (Table 2; Fig. 5). NWEI of L was estimated to be between 1.00 and 2.22 because eight midges probed multiple times before feeding to repletion, and four individuals transitioned between L and M multiple times during the same probe. The current type and Ri level combinations used to record the waveforms had a significant effect on the NWEI for L (LTR_(7)_ = 17.13, *P* = 0.017), and pairwise comparisons showed that NWEI of L was 2.22 ± 0.507-fold higher when using DC 10^8^ Ω compared with DC 10^10^ Ω (*Z* = 3.49, *P* = 0.011). On average, midges spent between 83.7 s and 133.9 s performing L (WDI), with each episode lasting between 42.7 s and 83.7 s (WDEI). However, the first episode of L (DurFrs) was generally longer than the average duration of each event (WDEI).

**Fig. 7:**
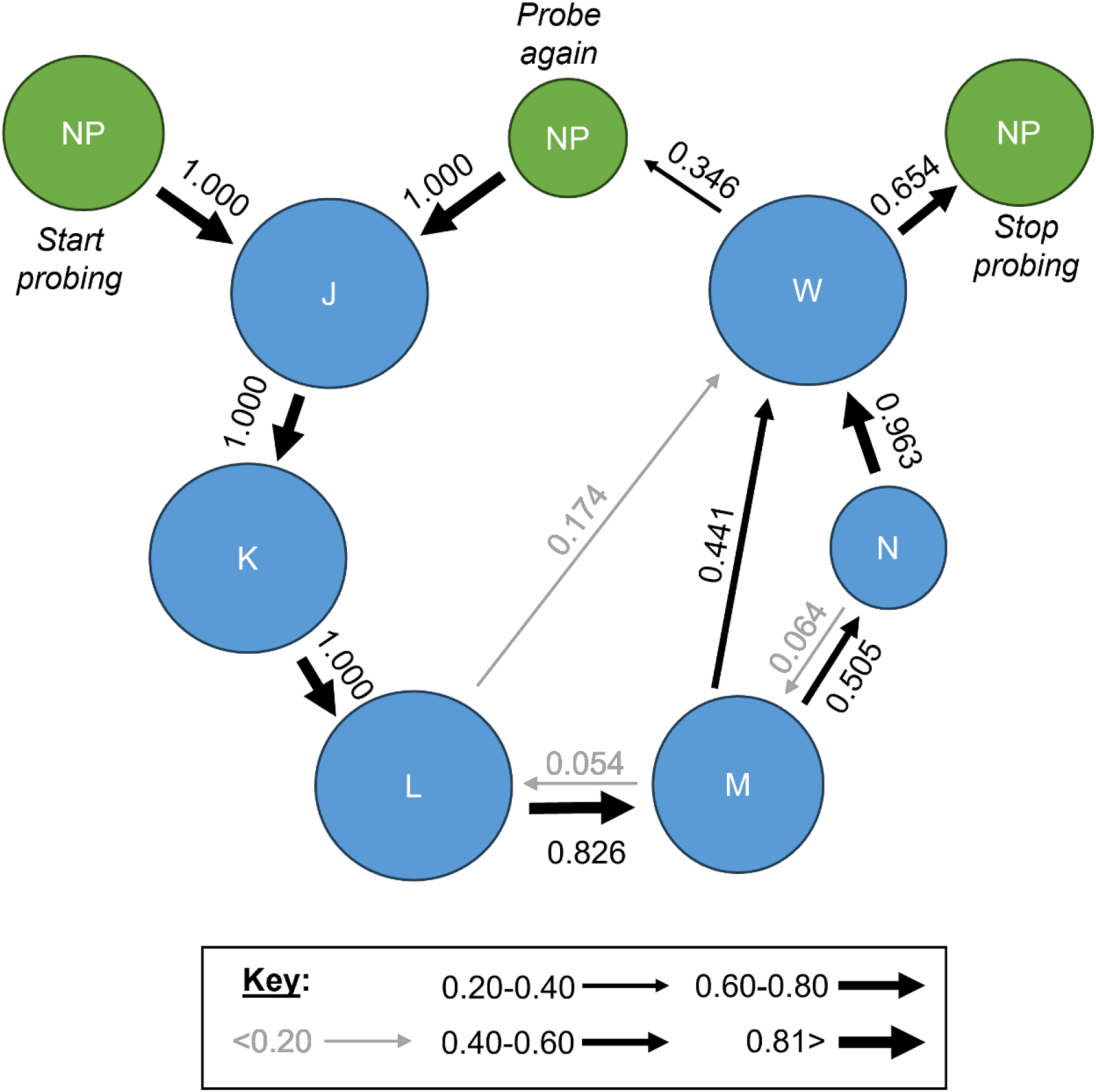
Kinetogram showing the probability of transitioning between waveforms. The probability of transitioning between *Culicoides sonorensis* waveforms during a hypothetical probe before feeding to repletion. The arrow width, as shown in the key, indicates the probability of transitioning between each waveform. Circle size represents the number of waveforms events (i.e., 172 for NP, 109 for L, 92 for M, 48 for N, 68 for R, and 104 for J, K, and W). Transitions happening randomly and nonrandomly according to the exact binomial test are shown in gray and black, respectively (i.e., the true probability of the transition is greater than 0.14 (1/7) if shown in black, indicating that the transition did not occur by chance)

Any painful sensations during an EPG session were most often reported by hosts during early L, usually near the end of the voltage dip following K (approximately 8–15 s into the probe). Some hosts reported intermittent pain throughout L that stopped 20–130 s into the probe, while others reported never feeling pain. The midges were observed periodically moving their heads, body, and antennae during L, partially inserting and withdrawing their mouthparts, and occasionally performing posterior grooming (Additional Files 2, 4–7: Supplementary Videos S1, S3–S6). Partial mouthpart withdrawal was also correlated with voltage dips during L, and partial re-insertion with a return to the predominant voltage level for L. Sometimes continued penetration of the mouthparts into the skin was observed during the voltage dip that followed K, further supporting that the voltage level was correlated with the depth of the mouthparts in the skin. In one instance, the head and antennae movements during L appeared to be correlated with waveform types containing large peaks (i.e., L1, L2, and L3). No blood was observed in the alimentary canal when midges were interrupted during L (Table 3; Fig. 7), indicating that L is not correlated with blood ingestion. Occasionally, a single excretory droplet or a slightly swollen abdomen was observed on video during L but only if waveform family M was present in a previous probe.

#### Family M

**Fig. 8:**
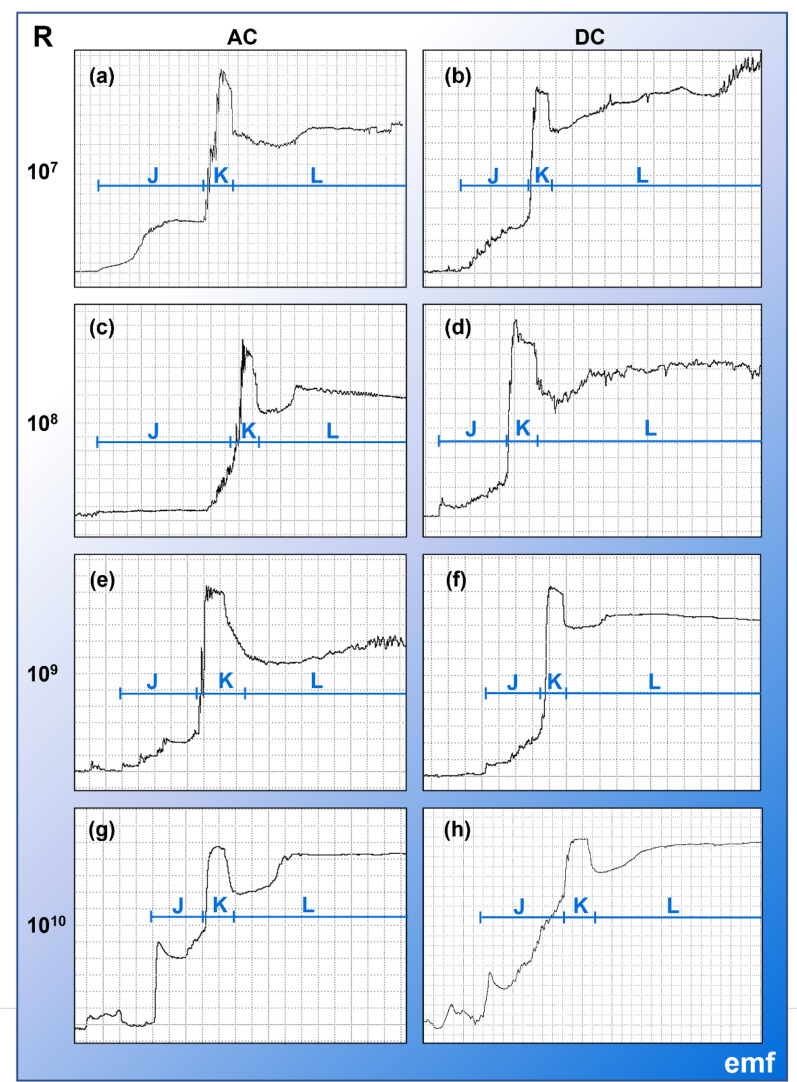
Comparison of the appearance of waveform families J, K, and early L across treatment groups. Comparison of *Culicoides sonorensis* waveform families J, K, and early L when recorded at different input resistances (10^7^,10^8^, 10^9^, 10^10^ ohms) and current types (alternating current, AC; direct current, DC) combinations. Emphasis on electromotive forces (emf) versus biological resistance (R) components at the different setting combinations is indicated by the color gradient in the background. Time scales are 1 second per division, and Windaq gains are: 64x (**a**); 16x (**b**); 32x (**c**); 32x (**d**); 16x (**e**); 8x (**f**); 8x (**g**); 8x (**h**)

M occurred at a similar or slightly higher voltage level than L and comprised peaks and occasional expanses that were classified into five types: M1, M2, M3, M4, and M5 (Figs. 3, 4; Table 1). On average, NumPrFrs for M in *C. sonorensis* was between 4.00 and 5.67 (Table 2) because not all probes contained M (Figs. 8, 4, 5). For most midges, NumPrFrs was 4, but for some individuals, it was 9, 14, and in one case, 19 (extreme outlier), indicating that some midges (i.e., those who probed multiple times before feeding to repletion) performed numerous other waveforms before performing M. NWEI for M showed that midges performed M 1.00–1.67 times on average, and WDI for M indicated that midges spent on average 287–371 s performing M. Each individual event of M lasted between 227 s and 343 s (WDEI), with the duration of the first event of M performed by each midge being between 235 s and 382 s (DurFrs).

The midges were predominantly motionless during M, except for abdominal changes, caudal segment movements, and the release of abundant excretory droplets (Additional Files 5, 8, 9: Supplementary Videos S4, S7, S8). Occasionally intermittent body movements and posterior grooming were also observed. Hosts sometimes reported painful sensations near the middle of M or at the end shortly before waveform W. Blood was present in the alimentary canal of all midges that were interrupted during M for dissection (Table 3, Fig. 7), confirming that M is correlated with blood ingestion in *C. sonorensis*.

#### Family N

**Fig. 9:**
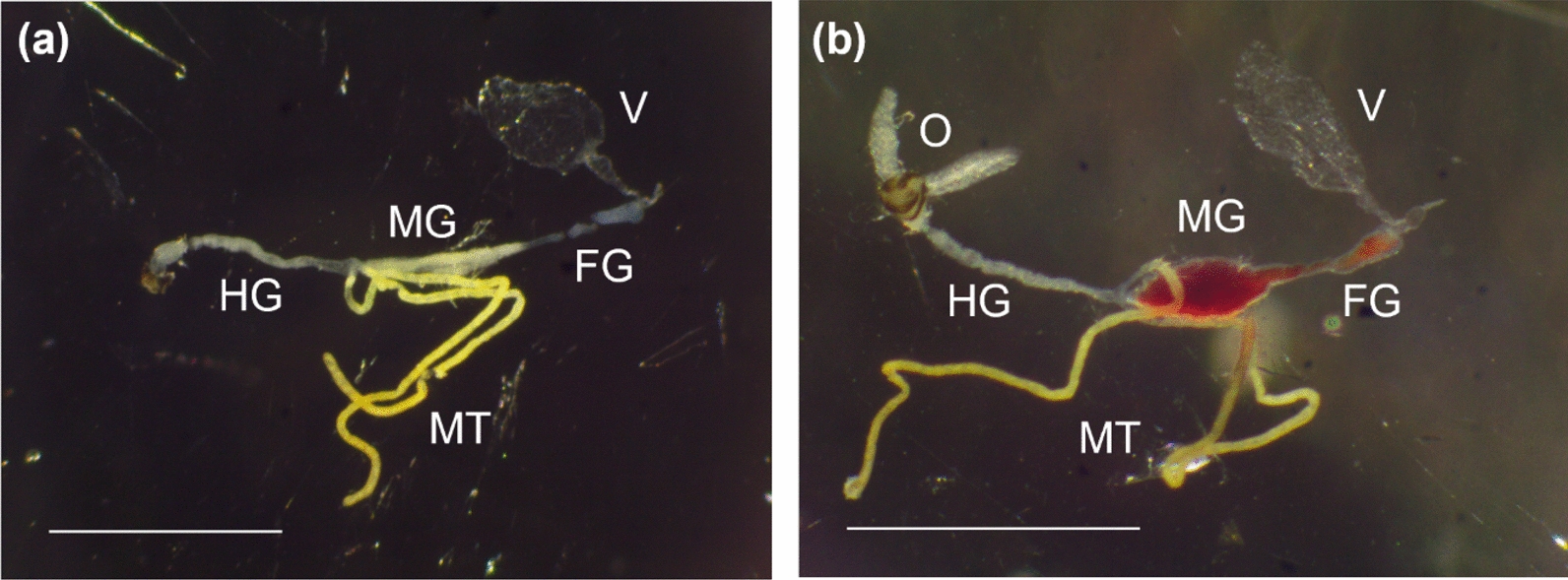
Representative images of dissected guts from interruption experiments. Dissected guts from *Culicoides sonorensis* that were interrupted 23 s into waveform family L (**a**) and 35 s into waveform family M (**b**). Scale bars indicate 1 mm. Abbreviations: FG, foregut; HG, hindgut; MT, Malpighian tubules; MG, midgut; O, ovaries; V, ventriculus

N only occurred in singular and final midge probes and comprised low-frequency skewed peaks and long expanses, usually at a similar voltage level as M (Figs. 3, 9, 4). N was divided into two types, N1 and N2 (Table 1). When performed, N usually occurred in a single episode at the end of the probe but occasionally was interspersed with M, as reflected by NEWI values for N near one and NumPrFrs for N between 5.00 and 9.75, on average (Table 2). On average, midges that performed N spent 67.1–193 s in this waveform (WDI), with each event of N lasting between 55 s and 193 s (WDEI). DurFrs for N was similar to WDEI.

The midges were mostly still during N, except for caudal segment movements, the release of excretory droplets, and, occasionally, intermittent body rocking or movements (Additional File 10: Supplementary Video S9). In addition, their abdomens were always swollen and red. Hosts usually could not feel the midge during N and were often unsure if the midge was still probing or not. However, on one occasion, the host reported feeling pinprick sensations. Interruption experiments were not performed during N because the abdomen was already visibly full of blood before N occurred.

#### Family W

In *C. sonorensis*, W appeared as a sudden and immediate drop back to the NP baseline (Figs. 3, 4; Table 1), occasionally with a peak, or a slight stairstep near the middle. Owing to the brief and nonrepetitive nature of W, statistical analysis and frequency calculations were not performed. During W, midges rapidly withdrew their mouthparts from the host, often accompanied by a slight body jerk or hop (Additional Files 5, 6, 9, 10: Supplementary Videos S4, S5, S8, S9). After W, usually the maxillary palps closed immediately, but palps of non-replete midges would sometimes remain slightly open until the next probe.

## Discussion

In this study, EPG procedures for *C. sonorensis* were developed and optimized on the basis of the procedures for mosquitoes and other insects. Owing to the minute size of biting midges, very fine gold wire was used as the insect electrode to permit natural movements and behaviors during EPG. The wiring procedure allowed for recording clear and interpretable waveforms that were suitable for characterization and analysis, revealing that *C. sonorensis* generates waveform families J, K, L (types 1, 2, 3, 4, and 5), M (types 1, 2, 3, 4, and 5), N (types 1 and 2), and W when probing on hands. These waveforms were previously reported in mosquitoes except for L4, L5, and M5 [5, 6]. As hypothesized, there were distinct differences in the appearance of the waveforms generated by midges versus mosquitoes. Still, a similar sequence of waveforms suggests a conserved series of feeding behaviors despite anatomical differences in these insects.

The waveforms from *C. sonorensis* were identified and described on the basis of their relative voltage levels, general amplitudes, peak shapes, and peak frequencies (Table 1). The probability of transitioning between the waveforms (Fig. 5), as well as statistical analysis of the count and duration of each waveform (Table 2), demonstrated plasticity in waveforms L, M, and N. Slight differences in the appearance of these waveforms across Ri levels (Figs. 3, 4, 6) were used to infer the electrical origins of each waveform. R components are more visible at lower Ri levels, and emf components are more pronounced at higher Ri levels [17]. Thus, the optimal Ri levels for studying each waveform individually and collectively were identified (Table 1). When the Ri level matches the inherent resistance of the arthropod (Ra), the waveforms contain an equal mix of emf and R components and provide the most information [17]. Our results indicate that the Ra for *C. sonorensis* is between 10^8^ and 10^9^ Ω. However, fewer midges probed the offered hand when Ri = 10^9^ Ω was used in combination with AC (Supplementary Fig. S2b), leading to the recommendation that Ri = 10^8^ Ω using an applied DC signal of 75 mV be used for future EPG studies of *C. sonorensis*; however, the voltage may need to be adjusted slightly when using different hosts to increase the signal-to-noise ratio [18]. Probing rates also varied between hosts (Supplementary Fig. S2a), demonstrating variability in attractiveness and palatability among humans to *C. sonorensis,* as previously documented for* Culicoides impunctatus* [19, 20]. Host visual observations and sensations, combined with video recordings of *C. sonorensis* during EPG sessions, provided insight into the behaviors corresponding to each waveform family (Additional Files 2–10: Supplementary Videos S1–S9) and revealed *C. sonorensis* performing putative grooming behaviors that are associated with the release of sex pheromones initiated by the presence of blood volatiles previously described in *Culicoides nubeculosus* [21]. Interruption experiments confirmed that blood ingestion is correlated with waveform family M. The observations and results presented here, in combination with the electrical origins of the waveforms, previous EPG studies, and biting midge literature, support the following interpretations of the waveforms.

J in *C. sonorensis* is hypothesized to correspond to salivation on the skin surface, as it resembles J in *Ae. aegypti* and *Cx. tarsalis* and has similar electrical origins [5, 6]. The emf-dominated slight voltage rise during J (Fig. 6) for *C. sonorensis* is consistent with wet mosquito stylets contacting the skin [5]. However, in video recordings, J in *C. sonorensis* appears to correlate with initial stylet penetration into host tissue, which is consistent with the R-dominated arch during J immediately before K. Stylet penetration during J was not previously reported in mosquitoes, possibly due to visual masking by the labellae or because waveform/video synchronization may not be perfectly aligned. These observations may indicate penetration of non-living epidermal tissues beginning during J and penetration of highly conductive living dermal tissues during K in both insects. Surface salivation has not been reported in biting midges but is thought to mask the mosquito stylet insertion into host tissue [5]; a similar phenomenon may occur in biting midges, as they secrete many of the same salivary proteins as other blood-feeding insects [22]. Further investigation is needed to confirm the biological activities associated with J.

The prominent voltage rise at the beginning of K (Fig. 6) is consistent with skin penetration and may occur once the stylets penetrate living, conductive dermal tissues. In many insects, the voltage level is correlated with the depth of the stylets inside host tissues [23]; videos of *C. sonorensis* during EPG support this (Additional Files 2, 5: Supplementary Videos S1, S4). Salivation likely occurs during penetration, as in mosquitoes [24], and may be responsible for the small R-dominated peaks visible in K at low Ri levels. Interestingly, the R-dominated peaks during K in *C. sonorensis* are less prominent and do not linger into L like mosquitoes [5, 6], indicating salivation or other behavioral differences during host tissue penetration between these insects. The plateau during *C. sonorensis* K has minimal prominent voltage peaks compared with mosquitoes [5, 6]; however, the biological origin is unknown.

L is proposed to represent preparation of the ingestion site and formation of a hematoma in *C. sonorensis*, which is analogous to the search for a blood vessel in host tissues by mosquitoes, and likely involves salivation as well [5, 24–26]. Both *Ae. aegypti* and *Cx. tarsalis* have large upward-going R-dominated peaks during K and early L that are hypothesized to correspond to salivation events [5, 6]. These spikes are absent from *C. sonorensis* waveforms. However, L1 and L2 look similar between biting midges and mosquitoes, suggesting that the large R-dominated upward-going spikes in mosquitoes are not salivation events or that salivation behaviors differ considerably between these species. Because biting midge mouthparts do not meander through host tissue similar to mosquito stylets [27, 28], the shape and frequency of L1, L2, and L3 peaks are more likely due to salivation than mouthpart movements.

L4 and L5 were not reported in mosquitoes. Flat expanses such as L4 were observed in mosquitoes but were included in other waveform types [5, 6]. Here, the flat expanses during L were classified as L4 because they were visible at intermediate Windaq compression levels (1–10), meeting the definition of a waveform type [6, 29]. L4 may represent brief pauses in salivation and mouthpart movements during ingestion site preparation (Additional File 5: Supplementary Video S4). The absence of L5 in mosquitoes combined with the high frequency of the small peaks suggests a sawing motion by midge mouthparts for lacerating host tissues [27, 28].

M was experimentally correlated with blood ingestion in *C. sonorensis*, as in mosquitoes [5, 6]. Salivation may occur during M as well [25, 30–32]. M in midges looks like M in mosquitoes, suggesting similar ingestion behaviors. Wayadande et al. [5] hypothesized that M1 corresponds to ingestion from a blood vessel, whereas M2 corresponds to salivation into a blood vessel. In *C. sonorensis,* M1 and M2 may represent ingestion from and salivation into the hematoma, respectively. However, hemophagous dipterans have separate salivary and food canals, making simultaneous ingestion and salivation possible [9, 33]. M3 often contains upward-going peaks as well as plateaus in *C. sonorensis* and mosquitoes [6] and may represent simultaneous salivation and ingestion. In both mosquitoes and *C. sonorensis*, M4 likely represented brief pauses in ingestion, during which occasional salivation events occur, as represented by sporadic upward-going peaks. M5 was not observed in mosquitoes. M5 only occurred near the end of *C. sonorensis* probes and may represent alternation between salivation, ingestion, and brief pauses as the midge nears repletion and/or the hematoma begins to clot.

Interestingly, there was no large R-dominated voltage rise at the beginning of M in *C. sonorensis* as in mosquitoes [5, 6], likely because biting midges do not feed directly from a highly conductive blood vessel. The frequency of peaks during M was lower in midges than in mosquitoes [5, 6], consistent with Kashin’s [24] observations that the peak frequency is lower during pool feeding than capillary feeding in *Ae. aegypti*.

The biological meaning of N is unknown, but may be a resting phase, as hypothesized in mosquitoes, since it only occurs after presumed repletion. However, the M to N transition was difficult to define in *C. sonorensis* compared with mosquitos. Like *Cx. tarsalis* [6], both N1 and N2 were generated by *C. sonorensis* when feeding on hands. N1 resembles M2 but with less defined peaks, and N2 resembles M4 but with much longer durations, so it is unclear if the biological activities occurring during M2/N1 and M4/N2 are similar. Interestingly, the appearance of M4/N2 in both mosquitoes and midges is consistent with putative resting (R1, R2) waveforms during slow-phase tick feeding [8]. Future correlation studies may show that it is more accurate to classify N as M.

On average, final/singular probes in this study were over 8 min in duration, but literature asserts that biting midges are very rapid feeders, with *C. sonorensis* taking 1–3 min to engorge [27, 34]. This discrepancy could be an effect of the EPG procedure, the strain of colony-reared biting midges used, the hosts, or other factors. To investigate the possibility that EPG caused the midges to probe abnormally long, untethered, fasted and unfasted midges were individually placed in glass vials, allowed access to a hand, and their probing duration determined using a laboratory timer. All the midges that probed (*n* = 10) did so for over 8 min (probing was artificially terminated after 11 min), except for one that fed to repletion in 4 min and 46 s, supporting that the EPG procedure itself did not affect the probing duration of *C. sonorensis* on the hands in this study.

This work sets a foundation and enables future investigations of the relationship between plasticity in *C. sonorensis* probing and ingestion behaviors, pathogen transmission, and host pathology. Animal models for EPG with *C. sonorensis*, such as the murine model developed for *Ae. aegypti* [35] will aid in this work and allow for a deeper understanding of *Culicoides*-host–pathogen interactions and facilitate novel insights into blood-feeding behavior. In addition, the hypotheses and speculation about the biological meaning of *C. sonorensis* waveforms presented here are a starting point for future correlation studies. The prevalence of these waveforms needs to be explored in other biting midge populations and species to determine how representative this dataset is, which employed midges that had been colonized for ~50 years. In addition, the effects of the biotic (age, strain, reproductive status, etc.) and abiotic conditions (lighting, temperature, time of day, etc.) employed in this study need to be comprehensively investigated to determine their effects on the probing and ingestion waveforms reported here.

## Conclusions

This *C. sonorensis* waveform library provides new insight into *Culicoides* probing and ingestion behaviors and sets the foundation for future EPG studies with biting midges. The six waveform families and nine types generated by *C. sonorensis* feeding on hands were characterized, and the optimal settings for studying each individually and collectively were determined. All the waveforms had previously been identified in mosquitoes, except for L4, L5, and M5. Ingestion was experimentally correlated with family M. Differences in probe durations, waveform types, and transition probabilities demonstrate that *C. sonorensis* does exhibit plasticity in probing behaviors. Overall, this investigation is expected to facilitate quantitative studies of the effects of various factors, such as pathogen infection, on the probing and ingestion behaviors of biting midges. This may lead to developing novel management strategies and a greater understanding of blood-feeding arthropod biology.

## Supplementary Information

Additional file1: Table S1. Control box gain and voltage settings used to record *C. sonorensis* probing on hands at each input resistancelevel. Table S2. Number of probes used to construct the *C. sonorensis* waveform library. Table S3. P-value matrix from multiple exact binomial tests for transition events between *C. sonorensis* waveform families. Supplementary Fig. S1. Boxplot showing the ages of *C. sonorensis *that contributed to the final dataset. Supplementary Fig. S2. The number of *C. sonorensis* that probed and engorged, probed but did not engorge, and did not probeoneach host andat each current type and Ri level combination.

Additional file 2. Video S1. Spreading of maxillary palps and penetration during J and K at half speed. Waveforms recorded at 75 mV AC, Ri=10^8^﻿ Ω.

Additional file 3: Video S2. Spreading of maxillary palps and penetration during J and K at half speed. Waveforms recorded at 75 mV AC, Ri=10^8^ Ω.

Additional file 4: Video S3. Spreading of maxillary palps, penetration, and slight head bobbing during J and K. Waveforms recorded at 50 mV AC, Ri=10^9^ Ω.

Additional file 5: Video S4. Spreading of maxillary palps, slight rocking, and penetration during J, K, and L, partial insertion and withdrawal of mouthparts during L, body movements and posterior grooming during L and M, slight abdominal swelling and withdrawal during M, and closing of the maxillary palps at W. Waveforms recorded at 75 mV DC, Ri=10^8^ Ω.

Additional file 6: Video S5. Slightly spread maxillary palps during NP between probes and penetration during J, K, and early L. Waveforms recorded at 75 mV AC, Ri=10^8^ Ω.

Additional file 7: Video S6. Head and antennae movements during L and lack of movement during M, showing the correlation of movements with voltage changes during L. Waveforms recorded at 75 mV DC, Ri=10^8^ Ω.

Additional file 8: Video S7. Abdominal swelling, caudal segment movements, and release of excretory droplets during M. Waveforms recorded at 75 mV DC, Ri=10^8^ Ω.

Additional file 9: Video S8. Abdominal changes and withdrawal during M, and closing of maxillary palps at W. Waveforms recorded at 75 mV AC, Ri=10^9^ Ω.

Additional file 10: Video S9. Caudal segment movements, excretory droplets, intermittent body movements, and withdrawal during N. Waveforms recorded at 75 mV DC, Ri=10^8^ Ω.

## Data Availability

Data supporting the main conclusions of this study are included in the manuscript.

## Abbreviations

AC: Alternating current
cm: Centimeter
DC: Direct current
DurFrs: Duration in seconds of the first event of the specified waveform
emf: Electromotive forces
EPG: Electropenetrography
Hz: Hertz
lx: Lux
mV: Millivolts
NP: Nonprobing baseline
NPi: Nonprobing initial
NPm: Nonprobing middle
NPf: Nonprobing final
NumPrFrs: Number of events to the first event of the specified waveform
NWEi: Number of waveform events by insect
NWEI: Number of waveform events per insect
Ra: Inherent resistance of the arthropod
R: Biological resistance
Ri: Input resistance
RH: Relative humidity
TBF: Time from the beginning of the file
WDEi: Waveform duration in seconds per event by insect
WDEI: Waveform duration in seconds per event per insect
WDi: Waveform duration in seconds by insect
WDI: Waveform duration in seconds per insect
x: Fold
Ω: Ohms
