# Supplementary material for: A waveform library for the study of probing and ingestion behaviors of Culicoides sonorensis using AC–DC electropenetrography
Source: Parasit Vectors. 2025 Jul 7;18:265. doi: 10.1186/s13071-025-06899-5 (PMC12235845; doi:10.1186/s13071-025-06899-5)
Supplement: Supplementary file 1 — Additional file1: Table S1. Control box gain and voltage settings used to record C. sonorensis probing on hands at each input resistancelevel. Table S2. Number of probes used to construct the C. sonorensis waveform library. Table S3. P-value matrix from multiple exact binomial tests for transition events between C. sonorensis waveform families. Supplementary Fig. S1. Boxplot showing the ages of C. sonorensis that contributed to the final dataset. Supplementary Fig. S2. The number of C. sonorensis that probed and engorged, probed but did not engorge, and did not probeoneach host andat each current type and Ri level combination. [file 13071_2025_6899_MOESM1_ESM.docx]

**Additional File 1**

**Table S1.** Control box gain and voltage settings used to record *Culicoides sonorensis* probing on human hands at each input resistance (Ri) level. AC, alternating current; DC, direct current; mV, millivolts; Ω, ohms; x, fold.

| Ri (Ω) | Voltage (mV) | AC Gain (x) | DC Gain (x) |
| --- | --- | --- | --- |
| 10^7^ | 150 | 40-120 | 20-115 |
| 10^8^ | 75 | 20-100 | 10-100 |
| 10^9^ | 50 | 2-60 | 5-100 |
| 10^10^ | 50 | 1-20 | 2-30 |

**Table S2.** Number of probes used to construct the *Culicoides sonorensis* waveform library. AC, alternating current; DC, direct current; Ri, input resistance; Ω, ohms.

| Current Type | Ri Level (Ω) | Probes preceding the last probe | Final/singular probes to repletion |
| --- | --- | --- | --- |
| AC | 10^7^ | 4 | 8 |
|  | 10^8^ | 2 | 8 |
|  | 10^9^ | 7 | 10 |
|  | 10^10^ | 5 | 9 |
| DC | 10^7^ | 6 | 9 |
|  | 10^8^ | 10 | 9 |
|  | 10^9^ | 2 | 9 |
|  | 10^10^ | 0 | 8 |

**Table S3.** P-value matrix from multiple exact binomial tests for transition events between *Culicoides sonorensis* waveform families. **P* ≤ 0.05 indicates that the true probability of the transition is greater than 0.125, meaning the transition is not random. NPi, initial non-probing event (before probing); NPm, non-probing events between probes; NPf, final non-probing event (after probing is complete/the inset has fed to repletion); *P*, P-value.

|  | To NPi | To J | To K | To L | To M | To N | To W | To NPm | To NPf |
| --- | --- | --- | --- | --- | --- | --- | --- | --- | --- |
| From NPi | 1.000 | 0.000* | 1.000 | 1.000 | 1.000 | 1.000 | 1.000 | 1.000 | 1.000 |
| From J | 1.000 | 1.000 | 0.000* | 1.000 | 1.000 | 1.000 | 1.000 | 1.000 | 1.000 |
| From K | 1.000 | 1.000 | 1.000 | 0.000* | 1.000 | 1.000 | 1.000 | 1.000 | 1.000 |
| From L | 1.000 | 1.000 | 1.000 | 1.000 | 0.000* | 1.000 | 0.083 | 1.000 | 1.000 |
| From M | 1.000 | 1.000 | 1.000 | 0.993 | 1.000 | 0.000* | 0.000* | 1.000 | 1.000 |
| From N | 1.000 | 1.000 | 1.000 | 1.000 | 0.944 | 1.000 | 0.000* | 1.000 | 1.000 |
| From W | 1.000 | 1.000 | 1.000 | 1.000 | 1.000 | 1.000 | 1.000 | 0.000* | 0.000* |
| From NPm | 1.000 | 0.000* | 1.000 | 1.000 | 1.000 | 1.000 | 1.000 | 1.000 | 1.000 |

*Statistically significant.

**
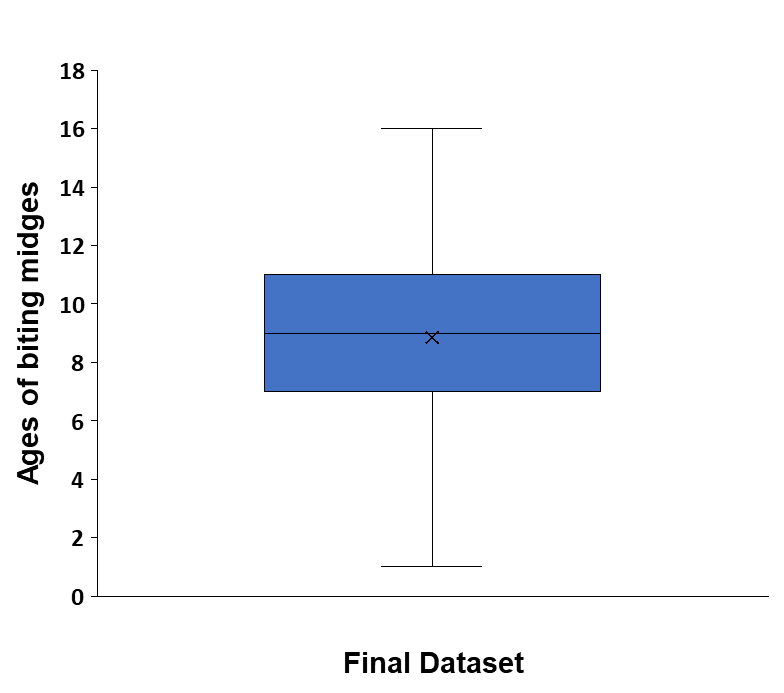
**

**Fig. S1**. Boxplot showing the ages of *Culicoides sonorensis* that contributed to the final dataset. Newly emerged adults (1-3 days post-exclusion) were initially utilized in this study, but they failed to probe and engorge at similar rates as previously observed by USDA Insectary personnel for other studies (*per comm.* W. Yarnell). In addition, newly emerged adults were not consistently available when hosts were. Therefore, midges of various ages were employed. The central box shows the interquartile range with the whiskers extending to the maximum and minimum. The mean age is indicated by the “X.”

**
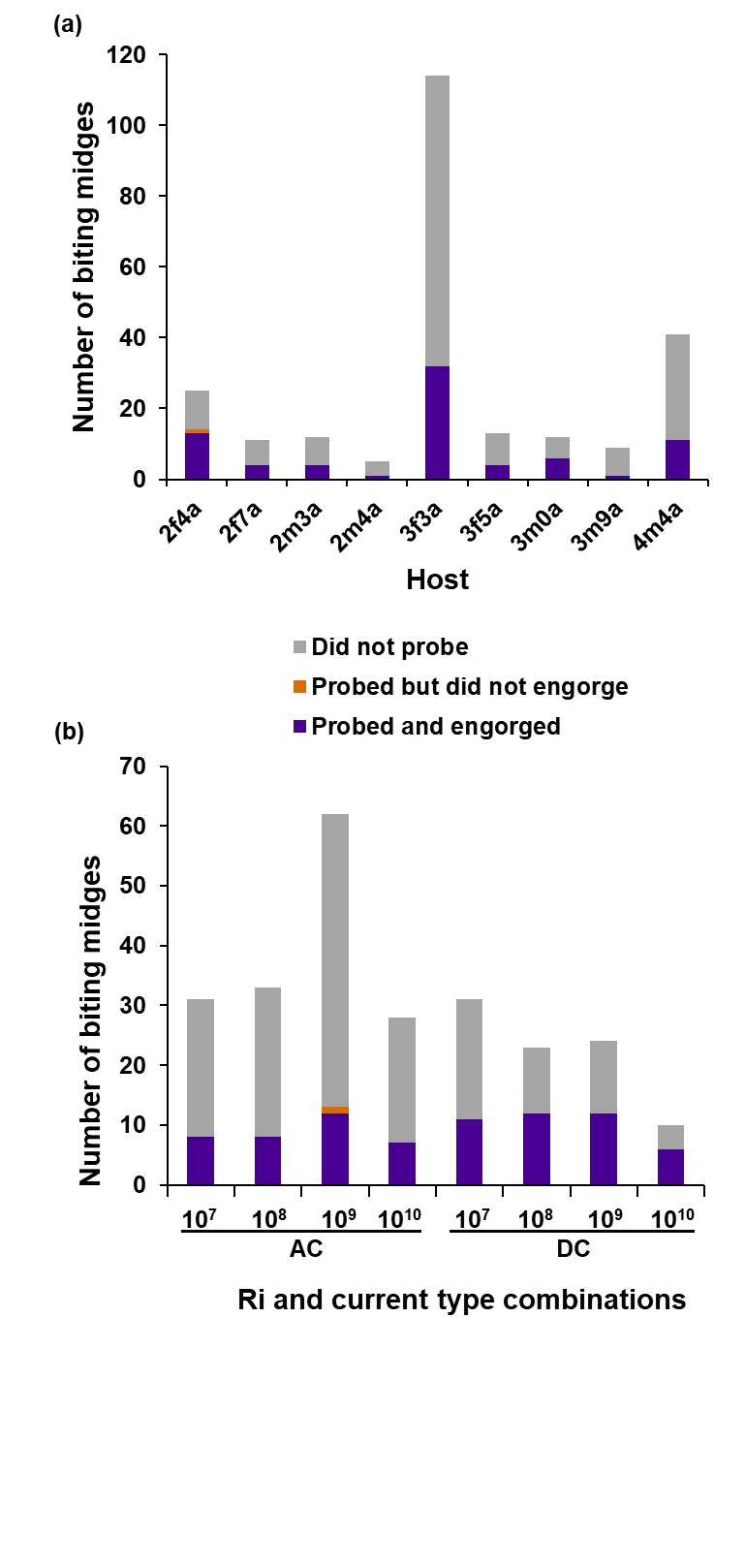
**

**Fig. S2.** The number of *Culicoides sonorensis* that probed and engorged (purple), probed but did not engorge (orange), and did not probe (gray) on (**a**) each human host and (**b**) at each current type and Ri level combination. Biting midges were exposed to a human hand for 10 min before switching to the next insect. Insects were screened until 8-10 useable EPG recordings at each treatment combination were achieved. Human hosts were scheduled based on their availability. Hosts that did not get bitten tended not to make themselves available for additional EPG sessions, so vastly different numbers of insects were screened on each host. Host identifiers indicate the age and gender of the host (i.e., 3f3a was the first 33-year-old female to enroll in the study). Note that considerably more insects were required to generate 8-10 usable EPG recordings at AC 10^9^ Ohms than at the other settings. In addition, the greatest proportion of insects fed to repletion on host 2f4a and the least on host 3m9a, demonstrating the variability in attractiveness and palatability of the human subjects. AC, alternating current; DC, direct current; EPG, electropenetrography; Ri, input resistance.
